# Right medium-sized vestibular schwannoma with trigeminal neuralgia post-fractionated radiotherapy

**DOI:** 10.3171/2021.7.FOCVID21112

**Published:** 2021-10-01

**Authors:** Kunal Vakharia, Anthony L. Mikula, Ashley M. Nassiri, Colin L. W. Driscoll, Michael J. Link

**Affiliations:** Departments of 1 Neurological Surgery and; 2 Otolaryngology, Mayo Clinic, Rochester, Minnesota

**Keywords:** vestibular schwannoma, trigeminal neuralgia, radiotherapy

## Abstract

A patient with trigeminal neuralgia secondary to a vestibular schwannoma underwent fractionated radiotherapy without relief of her pain. She was then effectively treated with microsurgical resection of her tumor. Early identification of the lower cranial nerves and the origin of the facial and vestibulocochlear nerves is key to determining the operative corridors for vestibular schwannoma resection. To effectively treat trigeminal neuralgia, the trigeminal nerve root entry zone and motor branch are clearly identified and decompressed. Fractioned radiotherapy does not effectively treat trigeminal neuralgia secondary to vestibular schwannoma compression.

The video can be found here: https://stream.cadmore.media/r10.3171/2021.7.FOCVID21112

## Transcript

### 0:22 Description of Patient.

Here we describe the resection of a right-sided medium vestibular schwannoma with trigeminal neuralgia status post-fractionated radiotherapy. Patient is a 51-year-old female with right-sided V2 trigeminal pain who was found to have a vestibular schwannoma that underwent fractionated radiotherapy with 48.6 Gy in 27 fractions over a 2-month period at an outside institution. She had sharp, persistent, lightening-type pain 6 months after radiotherapy.

On exam she had reduced pinprick in V1–V2 down the angle of the jaw on the right side. Her facial nerve was intact with no overt signs of vestibulopathy. Preoperative audiogram demonstrated moderate hearing loss in the right ear, with > 25 dB of hearing loss in that ear.^1^

Axial T1 contrast-enhanced MRIs demonstrate an homogenously enhancing tumor that extends all the way to the fundus. Axial FIESTA imaging demonstrates that the tumor is compressing the trigeminal nerve root entry zone and distorting the pons.^2^

Although the patient had recently been treated with fractioned radiotherapy, her trigeminal pain had persisted and worsened over several months. And thus, our rationale was to intervene at this point to give her the best chance of pain relief, especially because she had failed medical management.^3^

The patient was positioned in the lateral decubitus position, and the patient’s head was placed in a Mayfield head holder. An operative microscope as well as neuromonitoring for cranial nerves 5, 7, and 11 were used, although a good brainstem auditory evoked response could not be obtained in the right ear.

Key surgical steps include making sure the anterior and inferior edge of the craniotomy is extended all the way down to the edge of the sigmoid sinus to allow access to the lower cranial nerves. In addition, lower cranial nerves should be identified early on during the cisternal dissection, and an infraflocular approach can be used to identify the facial nerve. Identification and decompression of the trigeminal nerve root entry zone is key to treating trigeminal neuralgia.

### 2:25 Draining CSF From the Cisterns.

After the durotomy was performed, the CSF drainage from the cisterns allowed relaxation of the remainder of the cerebellum, and then the remainder of the durotomy is opened. Surgicel is used to line the cerebellum prior to the operation. Arachnoid dissection around the lower cranial nerves is done and the accessory nerve is identified early on.

### 2:45 Arachnoid Dissection Around PICA.

Arachnoid dissection is then carried around PICA, and the lower cranial nerves, including 9, 10, and 11, are identified. When the petrosal vein is superficial and it seems to be tethering the cerebellum, we do not hesitate to take it, as is routinely done for microvascular decompression. Peritumoral arachnoid dissection is then performed along the posterior aspect of the tumor to identify the planes along the cerebellum. After the tumor capsule is stimulated and no facial nerve is identified, the tumor capsule is coagulated.

### 3:18 Early Internal Debulking of Tumor.

A window is cut into the tumor all the way to the porus acusticus to allow for early internal debulking and maneuverability around the edges of the tumor. An ultrasonic aspirator is used to debulk inside the tumor to allow for more efficient manipulation of the tumor. Superior dissection is carried around the tumor capsule after the seventh nerve is stimulated and the course of the seventh nerve is identified. Here you can see that we’re dissecting tumor off of the cerebellar surface, and this is significantly harder secondary to the fact that the patient had prior radiotherapy.

Dissection is then carried along the posterior edge of the capsule and the proximal vestibulocochlear nerve is identified where the nerve splays out. It became apparent that we were not going to achieve the goals of the operation and save the eighth nerve, largely because of the tumor size, prior radiation, and the adherence of the tumor to the nerve. While we could identify it, the fact that it was running along the dorsal aspect of the tumor meant we would likely have to sacrifice it to safely dissect the tumor away from the facial nerve. Here we continue to dissect the tumor along the nerve.

### 4:46 Working to Identify Course of the Facial Nerve.

And at this point we work to identify where the facial nerve is as well. Neurostimulation helps at this point as well.^4^ We then stimulate along the eighth nerve to confirm its location and confirm the location of the seventh nerve as well. Then we debulk the tumor to allow for safer and easier manipulation without manipulation of the facial nerve and traction on the facial nerve. We then carry the dissection to the superior edge of the tumor after having identified the course of the facial nerve, and we dissect between the trigeminal nerve and the tumor capsule.

### 5:26 Untethering Superior Cerebellar Artery From Trigeminal Nerve.

We untether the superior cerebellar artery from the trigeminal nerve, and here we can see a view of the fourth cranial nerve, the superior cerebellar artery, and the trigeminal nerve. On the back end of the tumor, we identify where the cochlear nerve is as well as in the cistern deep we can see cranial nerve 6. We identify the splayed fascicles of cranial nerve 7 and stimulate to verify. Sharp dissection with microdissectors including a Rhoton 11 is used along the back edge of the tumor capsule, and after stimulation along the back edge of the tumor capsule to identify the exact fibers of the seventh nerve, this area is sharply incised and dissected off the back of the tumor capsule. Here you can see a combination of microinstruments used to develop this plane between the tumor and the seventh nerve.

### 6:18 Debulking Tumor to Limit Manipulation.

We debulk the tumor to limit manipulation of the seventh nerve, particularly around the porus acusticus given it is tethered to the bony region. After this is done, Gelfoam is placed along cranial nerve 6 and the cisterns.

### 6:39 Drilling of Porus Acusticus.

The dura is coagulated along the petrous bone, and an ultrasonic aspirator with a bone-cutting bit is used to drill the porus acusticus. During this process, the superior trough is created. The bone is then taken off the posterior porus acusticus and the dura is coagulated and then sharply incised and opened. At this point, the tumor is incised distally, and then microdissectors are used in a combination along the facial nerve from both proximal and distal to dissect the tumor off of the facial nerve. Here we can see that the tumor is in the internal auditory canal and is excised until normal neural structures are identified, including the cochlear nerve and facial nerve. Microdissectors, including the Cueva’s, are used to pull the tumor from the distal IAC after the correct plane is identified. Here we can see the beginning of the facial nerve that was originally stimulated and identified. The tumor is dissected from distal to proximal, and here you can see cranial nerve 7 and 8 identified in the distal porus acusticus. Microdissectors and round dissectors are used to sharply dissect the tumor and tumor capsule off the nerve again; being slightly more challenging, due to prior radiotherapy. The facial nerve is identified, and tumor is debulked off the nerve entry to the porus acusticus. Here you can see after stimulation we sharply incise that boundary, and an Apfelbaum mirror is used to inspect the distal internal auditory canal. The motor branch of the trigeminal nerve is stimulated and identified, and Teflon pledgets are used in typical fashion in a microvascular decompression to protect the trigeminal nerve root entry zone. Making sure we have adequate decompression at the entry zone is important, and here we see the anatomy of the superior cerebellar artery. Dural sealant is applied after the drilled surface of the bone is waxed.

### 9:10 Closure.

Postoperative axial T1 contrast-enhanced MRI demonstrates there is a gross-total resection. The patient had resolution of her trigeminal pain and was weaned off all medication. Her facial nerve was intact, but hearing was not preserved. Again, we see the postoperative FIESTA imaging demonstrating cranial nerve 5 and cranial nerve 7 after the decompression of the trigeminal nerve root entry zone.

There are several learning points from this case. First is early identification of the lower cranial nerves and the origin of the facial and vestibulocochlear nerves are key to determining the operative corridors for resection of vestibular schwannomas. Next is identifying the trigeminal nerve root entry zone and motor branch are key to dissecting out the trigeminal nerve and treating these patients’ trigeminal neuralgia. And finally, fractionated radiotherapy does not sufficiently and effectively treat trigeminal neuralgia/neuropathy secondary to compression of the nerve root by a vestibular schwannoma.^5^

## Disclosures

Dr. Nassiri received grants from Cochlear Americas outside the submitted work.

## Author Contributions

Primary surgeon: Link, Driscoll. Assistant surgeon: Vakharia, Nassiri. Editing and drafting the video and abstract: Link, Vakharia, Mikula. Critically revising the work: all authors. Reviewed submitted version of the work: all authors. Supervision: Link, Driscoll.

## References

[1] Lees KA Tombers NM Link MJ Natural history of sporadic vestibular schwannoma: a volumetric study of tumor growth Otolaryngol Head Neck Surg 2018 159 3 535 542 29685084 10.1177/0194599818770413

[2] Kamal N Reddy RK Kohli G The role of fast imaging employing steady-state acquisition (FIESTA) magnetic resonance imaging for assessment of delayed enhancement of fat graft packing on postoperative imaging after vestibular schwannoma surgery World Neurosurg 2018 114 e1066 e1072 29605696 10.1016/j.wneu.2018.03.147

[3] Cheng TM Cascino TL Onofrio BM Comprehensive study of diagnosis and treatment of trigeminal neuralgia secondary to tumors Neurology 1993 43 11 2298 2302 8232946 10.1212/wnl.43.11.2298

[4] Schmitt WR Daube JR Carlson ML Use of supramaximal stimulation to predict facial nerve outcomes following vestibular schwannoma microsurgery: results from a decade of experience J Neurosurg 2013 118 1 206 212 23140153 10.3171/2012.10.JNS12915

[5] Neff BA Carlson ML O’Byrne MM Van Gompel JJ Driscoll CLW Link MJ Trigeminal neuralgia and neuropathy in large sporadic vestibular schwannomas J Neurosurg 2017 127 5 992 999 28084915 10.3171/2016.9.JNS16515

